# Teaching broader impacts of science with undergraduate research

**DOI:** 10.1371/journal.pbio.2001318

**Published:** 2017-03-21

**Authors:** Kenneth C. Keiler, Kathy L. Jackson, Leslie Jaworski, David Lopatto, Sarah E. Ades

**Affiliations:** 1 Department of Biochemistry and Molecular Biology, Pennsylvania State University, State College, Pennsylvania, United States of America; 2 Teaching and Learning with Technology Research Group, Pennsylvania State University, State College, Pennsylvania, United States of America; 3 Department of Psychology, Grinnell College, Grinnell, Iowa, United States of America

## Abstract

Science plays an important role in most aspects of society, and scientists face ethical decisions as a routine part of their work, but science education frequently omits or segregates content related to ethics and broader impacts of science. Undergraduate research experiences have the potential to bridge traditional divides in education and provide a holistic view of science. In practice, these experiences can be inconsistent and may not provide the optimal learning environment. We developed a course that combines seminar and independent research elements to support student learning during undergraduate research, makes ethical and societal impacts of science clear by relating them to the students’ own research projects, and develops students’ ethical decision-making skills. Here, we describe the course and provide resources for developing a similar course.

The National Institutes of Health (NIH) has recognized that instruction in ethics is essential to provide a foundation of research integrity [1]. Ethics in this context includes responsible conduct of research, such as questions of research misconduct, data management, and standards regulating the use of humans and animals in research. The NIH recommends that ethics instruction also extend to “the scientist as a responsible member of society, contemporary ethical issues in biomedical research, and the environmental and societal impacts of scientific research” [1]. Recent debates surrounding the potential for human genome editing and genetically modified organisms in food highlight the importance of science in society and the need for scientists to understand ethical issues and become ethical actors.

Much of the focus of ethics education has been at the postbaccalaureate level, and many graduate programs require training in ethical conduct [2]. In contrast, most undergraduates receive little to no exposure to ethical issues related to science [3,4]. When ethics is taught, it is often as an unconnected add-on to a course or in a separate course [5]. This presentation can give students the impression that ethics is separate from science rather than an integral part of science [5,6]. An additional challenge is to provide students with the tools to “recognize, articulate, and resolve ethical challenges” [7] on their own, instead of simply teaching students that science can impact society and that research integrity is central to good science. To address this need, we developed a course, Communities of Practice in Biochemistry and Molecular Biology (COP), which integrates undergraduate independent research with key concepts in the field, ethical analysis, and the broader impacts of science on society.

## Structure of the course

COP facilitates student learning of ethical reasoning and awareness of the broader impacts of science on society by linking these topics to the students’ own primary research. Students perform research in the laboratory of a faculty member, and students from different laboratories working in similar areas meet together in a seminar format to discuss both the science and ethical issues related to the science. For example, one section of COP focuses on antibiotic development and antibiotic resistance. This section has had students from five different labs that work on antibiotic discovery, mechanisms of antibiotic action, development of antibiotic resistance, and detection of antibiotic-resistant bacteria. Scientific discussions focus on these topics. Ethics and policy discussions in this section (described in more detail below) have focused on drug approval, antibiotic husbandry, hospital practices, and health care rationing, in addition to research ethics. The structure of COP is designed to enhance undergraduate research experiences, teach basic ethical reasoning skills, and provide mentoring groups for students during their education.

## Communities of practice

A community of practice is a group of people with different levels of experience working toward the same goal. Participation in a community of practice provides superior learning for all levels of students [8]. Many research laboratories at universities function as communities of practice, even if the term is not widely employed. The goal of the research component of COP is for students to become an integral part of the community of practice in the research lab, as a means of learning about the particular area of expertise in the lab and broader lessons on how science is practiced. Our anecdotal experience suggested that some students engaged in independent research could successfully integrate into the laboratory community, but many others had difficulty making connections between their research and what they learned in lecture courses, and they frequently did not understand the significance of their work or how to communicate it. Formal assessments of undergraduate research have indicated that student gains are much stronger in technical skills and general understanding of what science is than in the higher order intellectual processes involved in doing research [9–12]. To solve this problem in COP, students from laboratories that address related research questions form a peer-learning group that meets once per week in a seminar format. In the seminar meetings, students make connections between their research and the body of scientific knowledge, and they investigate the practice of research, ethical issues related to the research, and public policies that are important for science.

The COP course structure provides two mentoring groups for each student—scientists at a variety of experience levels in the research laboratory and a peer group of undergraduate students in the seminar. Students take the course on an ongoing basis, allowing them to master skills over time and enabling peer mentoring of less experienced students by more experienced students in the course. This practice is in keeping with research demonstrating that students need more than a summer or 1–2 semesters of independent research to realize lasting gains [11,13,14].

## Integrating ethics

COP is designed to help students learn an analytical approach to ethical decision-making that they can apply to many different problems, instead of comprehensively covering ethics or bioethics. Students learn to identify the central ethical question in a situation, analyze the relevant facts that are known and what still needs to be researched, identify stakeholders, and apply bioethical principles to reach an ethically justified course of action. These skills are practiced using case studies drawn from the news or public health issues that are directly related to the students’ own research to help them make connections between the science they do in the laboratory and the world around them. These basic tools give students a general method to reach well-reasoned, justifiable conclusions about questions they will encounter in future careers in science or as scientifically literate citizens.

## Course elements

In our department, we have organized COP sections around a variety of themes: antibiotic development and resistance, eukaryotic cell biology, host–microbe interactions, RNA biology, structural biology, and eukaryotic gene regulation. Each section uses the same core elements, although the exact content is tailored to the research focus of the section. The approach used in COP can be applied to most scientific fields and interdisciplinary studies, and could be easily incorporated into most curricula. A general description of each element is described below, and a specific example of a COP semester from the antibiotic development and resistance section is provided in Box 1. Detailed course materials including specific learning objectives, teaching guides, student guides, and grading rubrics for those interested in adapting the course for their own use are provided as supporting information (S1 Text).

**Box 1. Example of a COP section—Antibiotics: Resistance and Development.**

We have offered the COP section on Antibiotics: Resistance and Development for six years, and five labs host students in this section. Research interests of the labs include development of new antibiotics, mechanisms of antibiotic resistance, and diagnostic tools for identification of resistant strains. The semester described below was focused on tuberculosis. This section had 17 students.

**Week 1:** Reviewed syllabus and watched segments from the documentary “Rx for Survival: Rise of the Superbugs” that focus on the fight against antibiotic-resistant tuberculosis in the slums of Lima, Peru.

**Week 2:** Online discussion of ethical issues raised by the documentary (there was no class meeting due to a holiday).

**Weeks 3 and 4:** Discussion of Andries et al. A diarylquinoline drug active on the ATP synthase of *Mycobacterium tuberculosis*. Science. 2005; 307: 223–7, a paper reporting the discovery of bedaquiline, the first new drug approved to treat tuberculosis in 40 years. Students discussed each figure in groups of 4–5, followed by a discussion of the figures by the entire class. This format promotes participation by shy or inexperienced students.

**Week 5:** Peer evaluation of research proposals. Students brought rough drafts to class and read and evaluated them using a rubric (S1 Text) in groups of 4–5. The final draft was due the following week.

**Week 6:** Ethics case study about whether to treat patients with latent tuberculosis infections. Students identified ethical issues and stakeholders and evaluated the best outcome based on different ethical frameworks.

**Weeks 7 and 8:** Discussion of Andries et al. Acquired resistance of *Mycobacterium tuberculosis* to bedaquiline. PLoS ONE. 2014; 10: e102135, a paper describing resistance to bedaquiline.

**Week 9:** Group presentations of proposed experiments based on the paper discussed during weeks 7 and 8. Groups of 4–5 students decided what was the next logical experimental question and proposed an experiment to address that question.

**Week 10:** Discussion of the accelerated approval process for bedaquiline by the Food and Drug Administration, based on articles from primary literature (Avorn. Approval of a Tuberculosis Drug Based on a Paradoxical Surrogate Measure. JAMA. 2013; 309: 1349–1350 and Cox and Laessig. FDA Approval of Bedaquiline—the Benefit-Risk Balance for Drug-Resistant Tuberculosis. N Engl J Med. 2014; 371: 689–91).

**Week 11:** Debate: groups of students took positions of different stakeholders in the drug approval process and debated the benefits and risks of approving bedaquiline through the accelerated approval process.

**Weeks 12–14:** Student research presentations.

### Research project

Each student is required to have his or her own research project and not work as an assistant or apprentice to a graduate student or more senior scientist. The individual research projects of COP students can be part of a larger project in the lab, as long as the experiments the student performs are well defined and can produce an interpretable result. These characteristics facilitate the scientific practice and communication components described below. We believe that students also develop a better sense of responsibility toward the project when they perceive it as their own and are therefore more likely to engage their own intellect instead of relying on what they are told by others in the lab. Our implementation of COP requires students to work in the laboratory for at least five hours per week. This number was chosen because it aligns with credit policies for independent research in the department.

### Proposal

At the beginning of each semester, students write a two-page proposal in the format of a National Science Foundation predoctoral application, describing their plans for the semester. The proposal ensures that students have a research plan and that they understand why they are doing the experiments they have proposed. The proposal must include a broader impacts statement, so students have to think about how their research will impact science and society. Students are provided with a guide for writing a proposal, including what content should be in the broader impacts statement and a rubric that is used for grading (S1 Text). One seminar meeting is devoted to peer editing research proposals before they are turned in.

### Ethics and policy

Ethics and public policy issues pervade science, but these disciplines are not taught in many undergraduate curricula. The goal of the ethics and policy units in COP is to expose students to some of the issues associated with their research and to provide them with analytical tools for ethical analysis. Students are introduced to an ethical decision-making process through a short lecture (S1 Text) and then practice this process through several case studies. See Box 1 for an example of how the ethics and policy topics relate to the scientific papers discussed in class. We use a framework for ethical decision-making described in Box 2 that is similar to one developed first by the Hastings Center on Bioethics and then expanded on by the Northwest Association for Biomedical Research (NWABR) [15,16]. This approach is straightforward and helps to limit the impact of personal biases that individuals bring to a problem. The approach does not require an in-depth background in philosophy, making it more accessible to instructors. Examples of case studies and tips for teaching ethical analysis are provided in the supporting information (S1 Text).

Box 2. Ethical decision-making*

1. Identify the ethical issue.
2. Analyze what facts are known, what still need to be researched, and what is not known.
3. Identify the stakeholders and their points of view.
4. Apply the bioethical principles (beneficence/non-maleficence, justice, autonomy) to reach an ethically justified course of action.

*Adapted from NWABR Ethics Primer [16].

### Scientific papers

The topics studied in research labs are often not directly covered in students’ course work so they must learn the relevant background for their research by finding information from the scientific literature and other sources. New students in the course have shown a heavy reliance on nonscientific web searches and Wikipedia to acquire information, and information from these sources is typically reported uncritically. They also have difficulty reading and understanding scientific papers. To enable the students to gather reliable information, we developed guides on sources of scientific information and on how to read a scientific paper (S1 Text). Students learn how to evaluate sources based on objective criteria, such as the use of peer review by journals, the funding source, and citations. Students also have to learn how to get information from primary research papers. The parts of a typical paper are explained, as well as different approaches to reading a paper. Several papers are then read and discussed in class over the course of the semester—typically three papers during a semester with two hours devoted to each. We write study guides (a sample is provided in supporting information, S1 Text) for each paper to direct students’ reading and help them focus on key points. This structured approach ensures that the students explore the background material underlying their research and helps them put their research into a larger scientific context.

### Research presentations

Students present their research progress at the end of the semester in either an oral report or a poster presentation. These presentations require students to reflect on their goals for the semester and to what extent they have been achieved. Because other students in the course are not in the same research lab, students must be able to describe their research question and background material to a nonspecialist audience. Each spring, the students present a poster at a university-wide undergraduate exhibition. These posters are designed to communicate with an intelligent but lay audience about the importance of the research and the major findings. The guides we give to students to help them prepare an oral report and a poster can be found in the supporting information (S1 Text). One class period is allocated for peer evaluation of poster presentations before the poster session.

## Preliminary assessment

**Fig 1:**
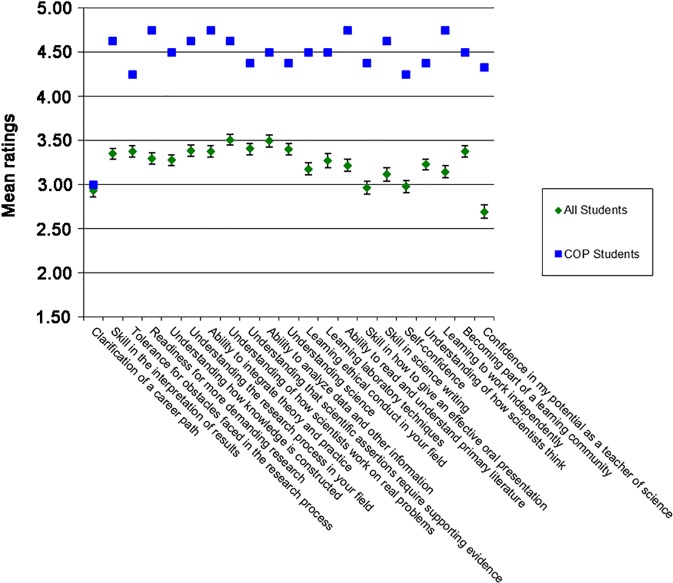
COP students perceive strong learning benefits. Mean ratings by students of perceived learning gains in the indicated items from the RISC surveys are shown (scale of 1–5, 1 meaning no experience or the student feels inexperienced and 5 meaning much experience or the student feels he or she has mastered the element). COP students’ ratings are shown in blue (*n* = 8) and ratings from all students who took the RISC survey between January 12, 2010 and January 8, 2014 are shown in green (*n* = 1,353, error bars represent 95% confidence intervals). The 21 items show a Cronbach’s Alpha of 0.97 taken on all data, both comparison and COP together. Data for “all students” is a convenience sample and is shown to provide a point of comparison for how COP students viewed their learning experience relative to a large sample of undergraduates from different universities taking disciplinary, interdisciplinary, integrated, and/or research-like science courses. Although this is not a matched group, it does demonstrate that COP students perceived important learning gains. A rigorous study with direct measures of student learning is planned.

A preliminary assessment of self-reported student learning gains in the COP section on antibiotic development and antibiotic resistance was obtained using the Research on Integrated Science Curriculum (RISC) survey instrument during the 2013 fall semester. RISC includes a precourse survey that collects student data on reasons for taking the course, level of experience, science attitudes, and learning style and a postcourse survey that parallels the precourse survey and adds questions on student estimates of their learning benefits and attitudes about science. As a comparison group, all student data over a four-year period that included the COP assessment were aggregated. The precourse surveys showed little difference between COP students and the comparison group with respect to their experiences in a variety of items associated with doing research. In the postcourse survey, COP students perceived higher benefits than the comparison group in most categories, including “learning ethical conduct in your field” (Fig 1, S1 Data). Although these data are measures of student attitudes and are not direct measures of student learning, they do suggest that students perceive significant benefits from the COP experience. COP student responses in a focus group interview were well aligned with findings of the RISC instrument. A COP student noted that “my critical thinking skills are so much better because I have to use them in this class.” Students also cite the ethics and policy units as broadening their view of the role of science in society and they appreciated being able to “explore the role, not just know the role” of science in society. When asked about the effectiveness of COP in increasing your ability to identify and analyze ethical issues in science, the student’s response that captured the range of comments best was “if it wasn’t for this class, I wouldn’t have the ability.” The initial assessment of student perceptions are encouraging, but a more complete assessment of key areas of COP, including ethical analysis, scientific thinking, and experimental design skills, that employs validated tools to directly measure student learning gains over several years will be required to determine if actual learning gains match student perceptions of those gains.

## Concluding remarks

COP provides students with a framework for their research that addresses many of the shortcomings noted with the traditional undergraduate research approach [9]. The integrated focus on the practice of science with the ethical and societal implications of the science allows students to see the connections between their research, the concepts they learn in lecture courses, and the broader impact of the science on society. Because students take COP for multiple semesters, they are able to develop skills over time and build strong a community with other students in COP and in their respective laboratories. This holistic view of research should improve the engagement of students who will need to understand and evaluate research as part of their jobs or lives even if they do not become scientists, in addition to those who intend to have a career in research.

## Supporting information

S1 Text Communities of Practice Course Materials. (PDF)

S1 Data Data from RISC survey. (XLSX)

## Abbreviations

COP: Communities of Practice in Biochemistry and Molecular Biology
NIH: National Institutes of Health
NWABR: Northwest Association for Biomedical Research
RISC: Research on Integrated Science Curriculum
