# Supplementary material for: Teaching broader impacts of science with undergraduate research
Source: PLoS Biol. 2017 Mar 21;15(3):e2001318. doi: 10.1371/journal.pbio.2001318 (PMC5360197; doi:10.1371/journal.pbio.2001318)
Supplement: S1 Text — (PDF) [file pbio.2001318.s001.pdf]

COMMUNITIES OF PRACTICE COURSE MATERIALS

1. Sample syllabus
2. Teaching ethics – guidelines and tips for teaching ethical reasoning in COP
3. Ethical reasoning introduction slides – slides used for introductory lecture
4. Sample ethics lesson plan
5. Sample ethics case study
6. Research proposal guide – material given to students to help them prepare a proposal
7. Proposal rubric
8. Proposal score sheet for peer review
9. Tips for teaching papers – suggestions for teaching papers from the primary literature in COP
10. Student guide for reading a paper – material given to students to help them read a research paper
11. Sample paper study guide
12. Advice on giving a research talk – material given to students to help them prepare an oral presentation on their research project
13. Presentation rubric
14. Presentation score sheet for peer evaluation
15. Poster guide – material given to students to help them prepare a poster on their research project

Materials can also be found online at https://sites.psu.edu/communities of practice/.

BMB 488 Communities of Practice in Biochemistry and Molecular Biology
Section 001: Antibiotics: Development and Resistance
2 credits

Instructors: ______________

Information: Course materials and information will be posted on the course web site.

Goals: The goal of BMB 488 Antibiotics: Development and Resistance is to achieve a holistic understanding of science through investigation of specific issues pertaining to antibiotic development and resistance. This goal will be attained by integrating the practice of primary research with the acquisition of key concepts of the discipline, including the philosophy of science, central concepts and details of an experimental system, and the implications of research on science policy and public health.

In this course, the students will:

1. Develop the ability to identify important scientific questions and pose them in a way that can be addressed experimentally.
   a. Students will be able to acquire sufficient information from reference sources, reviews, and primary literature to identify key questions in the field that have not been answered to their satisfaction.
   b. Students will be able to determine what new information is needed to address an open question, and what experiments are appropriate to test key aspects of the question.
2. Learn how to communicate scientific objectives, data, and conclusions.
   a. Students will write a 2 page project proposal at the beginning of the semester using NSF predoctoral fellowship guidelines.
   b. Students will present their research data in a poster session.
   c. At the end of the semester, students will give a short talk on their research.
   d. During the seminar, students will practice learning and communicating their critical thoughts in a peer group.
3. Develop the ability to gather, evaluate, and organize information outside a classroom setting.
   a. Students will learn how to identify appropriate sources for scientific knowledge and evaluate their relative quality.
   b. Students will learn how to read and interpret articles from the primary scientific literature. They will be able to identify the hypothesis, to critically evaluate the results and conclusions, and to relate information from an article to their own research.
   c. Students will learn and use the proper methods for citing sources.
4. Understand the societal implications of research.
   a. Students will be able to articulate the broader impacts of research projects related to the course section.
   b. Students will include a broader impact statement in their project proposal.

5. Acquire an understanding of ethical analysis as it applies to science, including the responsible conduct of research and ethical applications of science to society.
   a. Students will be able to identify ethical issues pertinent to an area of scientific research.
   b. Students will be able to identify stakeholders and their objectives.
   c. Students will be able to analyze the situation from different ethical perspectives.
6. Master research experimentation skills
   a. By working in a community of practice, students will learn from scientists with different levels of experience how to design, execute, and interpret experiments.

Research: Students will learn fundamental concepts of biology and experimental design by acting as an independent primary researcher within a community of practice, a group of scientists from many levels of experience working toward a shared goal. A minimum of 5 hours/week doing primary research in the assigned laboratory is required.

- At the beginning of the semester, students will write a short, 2 page paper describing the goal of their research project, the hypothesis to be tested, and the experimental approach to be used. The research plan will be due ______.
- All students will present a poster at the Undergraduate Exhibition on _____. Posters may be group or individual projects.
- At the end of the semester, students will give oral presentations on their research.

Seminar:
The major focus of the seminar will be on scientific and public health issues related to antibiotic development and resistance, with an emphasis on how those issues relate to infectious disease. Students will direct this aspect of the course and, as a group, choose topics for investigation in consultation with the instructors. Potential topics are listed below and students are encouraged to suggest additional topics. Students will also learn how to gather information outside a classroom (how to evaluate the reliability of a source, what constitutes evidence as opposed to opinion) and how to synthesize and present material to their peers. At the end of the semester, students will make oral presentations on their research projects.

Grading:
Laboratory research 50%: the laboratory part of the grade will be based on productive participation in the community of practice, including following the relevant safety protocols and experimental procedures. This portion will be assessed by the supervising faculty member.
Seminar 50%: The seminar grade will be based on the student's preparation for and participation in the discussions in class (30% of the course grade). The written proposal and oral presentation will each constitute 10% of the course grade. Since part of the grade is based on participation, attendance in class is critical. If you cannot come to a meeting of the seminar due to a valid excuse such as a documented illness, you must contact one of the instructors as soon as possible, preferably before the missed class.

Discussion topics:

1. Public health, science policy and ethical issues relating to antibiotic use and infectious disease. Potential topics include:
   - acquisition and spread of antibiotic resistant
   - challenges for antibiotic treatment regimens
   - the role of pharmaceutical companies in antibiotic development
   - the purpose and effects of government regulations relating to antibiotics
   - the impact of agricultural antibiotic use
   - ethical laboratory practices, oversight, and concerns
   - clinical testing of antibiotics
   - paying for antibiotic research and development
   - chemical libraries and screening strategies for antibiotic development biological warfare, terrorism, and defense
   - coverage of infectious disease and antibiotics in the press
   - public health agencies' roles in detecting and prevent the spread of infectious diseases

2. Current research: the class will investigate the biology of antibiotic resistance and bacterial pathogens through reading review and research articles from the primary literature.

Schedule (may be revised during the semester):

Week 1	Course Instruduction & Ethics 1 (responsible conduct of research)
Week 2	Paper 1
Week 3	Paper 1
Week 4	Research Propsoal workshop
Week 5	Paper 2 (research proposals due)
Week 6	Paper 2
Week 7	Paper 3
Week 8	Paper 3
Week 9	Poster Preparation
Week 10	Poster presentation practice
Week 11	Ethics 2
Week 12	Ethics 2
Week 13	Student Research presentations
Week 14	Student Research presentations

COP Tips for Teaching Ethics

Ethical analysis, as we have taught it in COP, is a structured approach to analyzing a problem and developing an ethically justified course of action. A case study approach works very well in which students are presented with a situation to analyze. Cases can be taken from the news as well as from case study databases available online. Another option is to discuss a journal article related to ethics in the field of interest.

It is important to emphasize that ethical analysis is not based on gut feelings, religion, political views, the law, or professional codes of conduct. We have used a structured approach that is outlined below. It involves an analytical and systematic exploration of standards that determines an ethically-justified course of action.

Ethical analysis:

1. identify the central ethical issue(s) in the situation
2. identify relevant facts and unanswered questions
3. identify stakeholders and their objectives
4. collect more information needed to assess the situation
5. identify possible solutions/options for action
6. use bioethical frameworks* to examine the impact on each key stakeholder
    - beneficence (maximize good)
    - non-maleficence (minimize harm)
    - autonomy (respect for individual)
    - justice (equal treatment)
7. provide a justification of choice of action supported by facts and ethical considerations

* Other ethical frameworks can be used such as consequentialism (focus on consequences- maximize good), deontological ethics (focus on duty of a moral actor), virtue ethics, and care ethics.

When considering topics for an ethics unit, ethics related to scientific research can be divided into three main areas.

1. Extrinsic ethics associated with science
    - impact of scientific research and its results on society
2. Intrinsic factors in doing science
    - unbiased interpretation of results vs. model bias
    - choice of methods can skew results
3. Responsible conduct of research
    - plagiarism, falsification of data, misrepresenting results, etc.

An excellent resource for background information on ethics, ideas for how to integrate ethics into the science classroom, case studies and lesson plans is the Ethics Primer from the Northwest Association for Biomedical Research (NWABR) https://www.nwabr.org/teacher-center/ethics-primer#overview.

Other resources for case studies in ethics include:

- National Center for Case Study Teaching in Science http://sciencecases.lib.buffalo.edu/cs/
- Columbia University Responsible Conduct of Research Courses http://ccnmtl.columbia.edu/projects/rcr/index.html
- President's Commission for the Study of Bioethical Issues http://bioethics.gov/node/5204
- Exploring Bioethics, NIH Curriculum Supplement Series https://science.education.nih.gov/supplements/nih9/bioethics/guide/default.html
- Bioethics 101, NWABR https://www.nwabr.org/teacher-center/bioethics-101#lessons

Ethics should be distinguished from:

- *Self-interest*: ethical actions don’t always benefit doer
- *Legality*: unethical action is not always illegal
- *Professional codes*: often limited in scope
- *Religion*: personal set of beliefs that can differ from religion to religion.

What is ethics?

Analytical and systematic exploration of standards that determine right and wrong conduct

→ NOT gut instinct

Ethical decision-making - Points of View

1. What are the consequences of the action?
   - What will produce the most overall good?
2. What is the duty of an ethical actor?
   - Obey universal principles- don't lie, cheat, etc.
   - Intentions matter
3. What would a virtuous person do?
   - honesty, respect, etc.
4. How does the action affect others?
   - network of relationships

Bioethical Principles

- *Beneficence* – do good
- *Non-maleficence* – do not harm (or minimize harm)
- *Respect for persons, respect for self-determination* – autonomy
- *Justice* – treat others equitably, distribute burdens fairly

Rubric for Analysis of Situations

1. What are the relevant facts?
2. What are the ethical issues?
3. Who are the stakeholders and what are their objectives?
4. Is more information needed- codes of conduct, policy guidelines, scientific information, etc.?
5. What solutions are possible?
6. Analyze situation using the bioethical perspectives to determine how stakeholders will be affected by the different alternatives
7. Decide upon a well-informed, ethically justified course of action.

Sample Lesson Plan for Ethics Unit

Learning objectives:
Students will
- identify the central ethical issue.
- identify the stakeholders and evaluate the consequences for each according to the frameworks/principles of bioethics: beneficence/non-maleficence, autonomy, justice.
- discuss the ethical issue from the point of view of different stakeholders.
- practice making reasoned arguments with those who may hold opposing points of view.
- decide on a course of action based on facts and ethical considerations.

Class 1:
1. Students read a case study.
2. Define ethical issue.
3. Identify relevant facts.
4. Determine what additional information is needed to evaluate the possible solutions.
5. Identify stakeholders.

Activities 2-5 can be done in small groups. Once students have discussed the issues in their group, hold a class discussion in which individual groups report their findings.

Homework:
6. Divide students into groups representing major stakeholders. Students gather additional information needed to evaluate solutions to the ethical issue from the perspective of their assigned stakeholder.

Class 2:
7. Students assigned to the same stakeholder meet in groups to discuss the effect of different solutions to the ethical issue on their assigned stakeholder according to the bioethics principles. They then decide on an ethically justified solution from the perspective of their stakeholder.
8. Students will then assemble in new groups so that each group has one representative of each stakeholder. They then hold a discussion to arrive at an ethically justified solution taking into account the positions of each stakeholder.
9. As a class, discuss each group’s solution to the ethical issue and the ethical justification for the proposed course of action.

Ethics Unit: Health Care Workers and Testing for MRSA

Case Study:

You are on the infectious disease control board of a major American hospital. The trauma intensive care unit (TICU) has experienced two major episodes of increased methicillin-resistant *Staphylococcus aureus* (MRSA) incidence over the course of a year. The second episode is thought to have been caused by colonized health care workers (HCW). As a result of this latest episode your board is meeting to consider instituting a policy for testing HCW for MRSA at the hospital.

The latest episode of increased MRSA incidence occurred in a group of 19 burn victims admitted to the TICU following a fire at a local nightclub. The TICU was cleared of existing patients and turned into a special unit for the burn victims. Following your hospital's procedures, which are in compliance with the Society for Health Care Epidemiology guidelines, all 19 patients were tested for MRSA colonization within 72 h of admission. All patients tested negative. Patients were monitored every 3-5 days thereafter for colonization or infection. By day six, one patient had developed a MRSA infection. The next week, five patients were found to be colonized by MRSA and three had MRSA infections. During the following two weeks an additional patient developed an infection and another was found to be colonized. Overall 11 of the 19 patients (58%) had either MRSA colonizations or infections. Five of these cases involved serious blood stream infections or ventilator-associated pneumonia. In the third week of the outbreak, HCW who had come in contact with the burn patients were tested for MRSA. Of the 133 HCW tested, six (4%) were found to be colonized with MRSA. All colonized HCW were treated with a murpirocin nasal spray and chlorohexidine showers for 5 days. These treatments cleared the colonizations from the HCW according to nare cultures. Because your hospital does not currently have the capacity to do whole genome sequencing of outbreak strains, pulse field gel electrophoresis (PFGE) was used to type the strains. Seven patients and four HCW were found to carry clone A, while two patients and one HCW tested positive for clone B. The remaining two patients and HCW had three unrelated clones. Both clone A and clone B were the dominant clones found during the first MRSA outbreak.

You are very concerned about the possibility that personnel in your hospital could have inadvertently instigated the outbreak, despite the controls already in place including the requirement for gowns and gloves when entering the burn victim's rooms that was put in place after 72 h.

A number of questions about the outbreak and overall hospital policy also remain unanswered.

- Could the outbreak have been caused by contact with another patient (directly or indirectly) who refused testing upon admission?
- Were the cleaning measures sufficient when the ward was vacated for the burn patients? There was limited environmental testing, so the possibility of airborne transmission or via environmental contamination can't be eliminated.

You decide to do some research to find data on transmission of MRSA from colonized HCW to patients. A survey of the literature identifies several articles that have examined the possibility that colonized HCW transmit MRSA to patients, but yields conflicting results. There are cases that clearly tie outbreaks to HCW. However, a study that examined 191 available outbreak reports revealed that there was strong evidence tying the outbreak to a HCW in only 11 instances (5.7%). A different study concluded that 27 of 101 (25.5%) outbreak cases could be traced to HCW. Nevertheless, many authors concluded that routine testing of HCW is too costly and problematic to be used effectively. In addition, studies estimate that only approximately 5% of HCW are colonized and that colonization can be transient, leading to

questions about when to test HCW. The CDC currently recommends testing HCW only when epidemiological data suggest that a HCW is involved.

Several possible courses of action are under consideration and your board is open to new ideas.

1) Institute routine testing of all HCW who come in contact with high risk patients.
2) Limit testing of HCW to times of increased MRSA incidence only.
3) Limit testing to times of increased incidence only when transmission by HCW is suspected to be a major cause of the outbreak, i.e. transmission occurs despite control measures.
4) Limit testing only to HCW who have come into contact with patients testing positive for MRSA.

Members of the board raise several concerns about the potential courses of action.

- What would be the optimum timing and frequency of testing?
- Should contaminated workers be excluded from working with high risks patients and for how long?
- What should be done with HCW who have recurring colonization or whose colonization is not cleared by standard procedures?
- What is the psychological impact on HCW? Will HCW who test positive be stigmatized?
- What would the impact be on staffing if workers are removed from patient care responsibilities for testing positive?
- What would the financial impact be to replace HCW testing positive?
- Does the hospital have a moral obligation to test and treat families of HCW along with testing and cleaning their homes?
- Is it right to make testing mandatory? What should be done about HCW who refuse testing?
- Testing many HCW is expensive. Is the cost justifiable?

References:

Albrich WC, and Harbarth, S (2008) Health-care workers: source, vector, or victim of MRSA? Lancet 8:289-301

Ben-David, D, Mermel, LA, and Parenteau, S. (2008) Methicillin-resistant Staphylococcus aureus transmission: the possible importance of unrecognized health care worker carriage. Am J Infect Control 36:93-7.

Hawkins, G, Stewart, S, Baltchford, O, Reilly, J (2011) Should healthcare workers be screened routinely for methicillin-resistant Staphylococcus aureus? a review of the evidence. J Hos Inf 77:285-9.

Vonberg, RP, Stamm-Balderjahn, S, Hansen, S, et al. (2006) How often do asymptomatic healthcare workers cause methicillin-resistant Staphylococcus aureus outbreaks? A systematic evaluation. Infect Control Hosp Epidemiol 27:1123-7.

COP Research Proposal Guide

Scientists fund their research through grants from public agencies such as the National Institutes of Health (NIH) and the National Science Foundation (NSF) or private agencies such as the American Heart Association and the American Cancer Society. To get this funding, scientists must write a research proposal that will convince grant review panels made up of other scientists that their research project addresses a problem that is critically important and that they have a well-thought out research plan that will successfully address the important question. Scientists at all levels write research proposals, from graduate students entering a PhD program applying for a NSF predoctoral graduate research fellowship to senior scientists applying for a research grant to fund a major project in their laboratories.

For this course, you will write a research proposal that has all of the major components of “real” research proposals. This exercise will give you experience at writing proposals and help you organize your thoughts about your research projects. A research proposal consists of the Background and Significance, Specific Aims, and Research Design and Methods. NSF proposals also must have a separate Broader Impact statement. A basic overview of what goes into each part is given below.

Background and Significance:
In this section of the proposal you should briefly describe the background of the proposed research and why it is important. Start with a relatively broad statement about the area of research and its significance. Next give more specific background information about your research topic. Then point out the research problem that you will be addressing and clearly state the hypothesis for your research. A clear hypothesis shows that you have a well-defined research question and that you will be able to advance the field by addressing this question.

A critical part of the background and significance section is to convince anyone reading your proposal that your research is important and must be done.

Specific Aims:
The specific aims are brief statements describing the objectives of your research project, what you want to accomplish. They state the major avenues of investigation that you will use to test your hypothesis. In formal research proposals, scientists usually have three specific aims. It is fine to have just one aim for your semester project.

Research Design and Methods:
In this section you briefly describe the experiments that you will be doing for each specific aim and the predicted outcomes of these experiments.

Broader Impact:
In this section, you should give a brief statement about how your research will benefit society. Taxpayers fund government-supported research and scientists should be able to explain why their research merits taxpayer dollars. The same applies to privately- funded research whose funds come from donors supporting a specific cause.

COP Research Proposal Guide

To give you a better idea of what to put into your research proposal, examples of significance, major problem, and hypothesis statements are given below. Although they are separated here, they should be integrated into the background and significance section along with a brief overview of the background relevant to the proposal.

A sample of specific aims that would be in this research proposal is also given below. The specific aims in your research proposal can be in the format presented here.

Significance: A newly described infectious disease caused by bacteria X is a major threat because no known treatment has been yet been found. To identify targets for drug treatment, it is imperative that we understand the mechanisms that give rise to the highly toxic virulent cells.

Major problem: Individual cells of bacteria X can be in one of two states: a benign non-pathogenic state and a severely virulent state [1]. Cells in the virulent state are only small percentage of the overall population, however they are highly toxic [1,2]. The molecular signals that determine whether an individual cell switches to the virulent state are not well understood. Previous work indicates that cells in stationary phase cultures are essentially starved for nutrients, that a higher rate of virulence is seen in stationary phase cultures, and that the starvation stress response is activated in stationary phase [3]. Based on this evidence, the goal of the proposed research is to determine the role of the starvation stress response in the formation of virulent cells.

Hypothesis: Activation of the starvation stress response is a major contributor to the formation of virulent cells.

Specific Aims:
This hypothesis will be tested with the following specific aims:
1) Assess whether virulence cell production is decreased in cells in which the starvation stress response has been disabled.
2) Analyze the level of activity of the starvation stress response in individual cells to determine if the response is preferentially activated in those cells that become virulent.

criterion	missing or unacceptable	developing	accomplished	exemplary
Title	missing or inappropriate	lacks relevance or does not convey appropriate details	relevant and includes details about project	informative, succinct, includes details and context of project
Hypothesis	missing or unrelated to project	related to project but not stated as testable hypothesis OR overly simplistic	stated as testable hypothesis	stated as testable hypothesis including implications of possible outcomes
Background	missing or unrelated to project	only partially relevant OR explains only part of the proposed research OR no references	describes all information needed to understand proposed research, references included	concise and focused, all information needed to understand proposed research, references included
Significance	missing or unrelated to project	states that project is significant but lacks convincing reason why	explains rationale for project	provides convincing reason that project must be done
Specific Aims	missing or unrelated to project	describes experiments but not goals or hypothesis	states major goals of the project	clearly and succinctly states major goals of the project and how they relate to the hypothesis

criterion	missing or unacceptable	developing	accomplished	exemplary
Research Design and Methods	missing or unrelated to project	techniques described but controls or analysis are missing	describes experiments including controls and analysis	clearly and succinctly describes experiments, controls, analysis, how results will be interpreted, potential problems and alternative approaches
Broader Impact	missing or unrelated to project	describes importance of research but lacks benefits for society	explains how research will benefit society	concisely explains how research will benefit society, including ethical and policy implications

Reviewer Name:______________________ Proposal by:______________________

Title:

Hypothesis:

Background:

Significance:

Specific Aims:

Research Design and Methods:

Broader Impact:

COP Tips for Discussions of Papers from Primary Scientific Literature

Reading scientific articles for the first time is intimidating and overwhelming for some undergraduates. A key goal of COP is to help students learn how to read and understand papers from the primary literature. We have developed an approach that helps students break down an article into manageable pieces, guides their reading of a paper, and involves all students in a discussion of the paper.

General Tips:

- Select articles that relate to a particular theme so that students can build their knowledge over the course of the semester.
- Identify 1-3 main points that you want the students to understand from a paper, rather than trying to cover all of the intricacies of the research. This approach helps keep students from being overwhelmed by the amount of information and helps them focus on what is most important about the research.
- Provide students with a study guide for an article to give them a framework to understand the paper and to help them learn good reading techniques.
- Do not have a student or group of students present a paper to the class while the other students sit and listen. In our experience, the students not presenting do not engage in the paper and discussion, even if they are required to read the paper and ask questions.
- Let students discuss the paper in small groups, before having a discussion with the whole class. We have found that this approach helps to alleviate students' anxiety about speaking in class and lets them see that other students have questions as well. Students also are often able to work out an answer to a question together that they were unable to solve individually.

Choosing a paper:

- Choose papers that are well-written, not too complicated, and not too long (unless you can devote several class periods to the discussion).
- Provide students with a relevant review article or "perspectives" piece, if available, to help give them some context for the research.

Writing a Study Guide for a Paper:

Study guides are designed to help students identify the important pieces of information in an article. We typically ask separate questions for the Introduction, Results, and Discussion to help students understand what type of information they should get from each section.

- Students often do not know the meaning of technical words and scientific jargon, making paper reading more difficult. Ways to address this are to:
    - provide a glossary of terms with the study guide.
    - give students a list of terms to define that they must know to understand the article.
- To help students better understand figures and tables, have them diagram the experiments that were done. Alternatively ask them to annotate the figure/table to explain what data are shown and what techniques were used.
- A sample study guide can be found below.

How to orchestrate classroom discussions:

Having students work through parts of a paper in small groups before holding a discussion with the entire class is a very effective way of engaging everyone in the discussion of a paper. We

want students to be willing to talk and share their ideas, even if they are not sure of the answer. Small groups give more students a chance to contribute and decreases the barrier to talking. By working together, students can help each other understand the material. After working in small groups, the full class discussion brings out the main points and can also reveal shared misconceptions held by the students that need to be corrected.

- Use the questions posed in the paper study guide to organize your classroom discussion.
- Rather than discussing the whole paper at once, break it into parts. For each part, have students discuss the questions posed in the study guide in small groups. Once they have finished the small group discussions, hold a discussion with the full class. Representatives from different groups can explain their group's response to the questions.
- For the results section, we either cover each figure individually or combine related figures and tables for the small group discussions.
- Another option is to have each group diagram an experiment that was done for a figure on the board. You can then compare the diagrams to let the students see that there are different ways to depict an experiment and to help them identify aspects of an experiment that they might have missed.
- Be patient — it will take ~2 full classes to get through a paper with 5-8 figures.
- Once you have completed the discussion of an article, you can extend it further by having students pose the next logical experiment for the research project. Students can peer-evaluate experiments in a grant panel format and decide which experiments they think would be best to pursue.

COP Guide to Reading a Scientific Paper

Reading a scientific paper can be intimidating at first. However, most well-written papers use a structure that makes it easier to extract information and evaluate. Once you are familiar with the structure, you will know where to find key pieces of information.

Abstract: An abstract gives an overview of the research question, approach taken, and major conclusions. Use this information to help guide your reading of the paper. The abstract should tell you what the hypothesis is and why the work is important. Highlight the major claims and verify they are supported by the data once you have read the paper

Introduction: Introductions are usually structured like the funnel paragraph that you learned about when starting to write essays in grade school. They often start with a broad introduction to the importance of the research problem. The next paragraphs then give the necessary background information so that another scientist, who is not necessarily an expert on the subject, can understand what is going on. Many introductions conclude with a paragraph or two describing the major questions addressed in the paper and what the authors consider to be the major conclusions.

Results: The results present the experiments done to address the research question. In a well-written results section, the authors will describe what question an individual experiment addressed, give a brief overview of what was done, and then describe the data. Often, they give a brief description of their conclusions from the data. Make sure to look at the figure or table associated with the text. Some scientists even look at the figures first without reading the text to evaluate the data on their own. Don't skip reading the figure legends. They are just that, a guide to understanding the figure. In class we will go through the results section figure-by-figure (tables too).

Materials and Methods *(Procedures, etc.)*: This section should give sufficient information for another scientist to repeat the experiments in the paper. When you are new to reading papers, don't spend time on this section (and there is no need to read it from start to finish). You can refer to it if you have questions about how an experiment was done.

Discussion: The discussion section describes in detail the individual conclusions from the research. Many discussion sections open with another summary of the major question and conclusions of the paper. Authors will discuss their results in the context of what is known and what are the outstanding questions in the field. In some cases authors may propose a model based on their results.

Important questions to consider and highlight or note when reading a paper.

Abstract and Introduction:
- What are the key questions or hypotheses being addressed in the paper? What are the major conclusions of the paper in broad outlines?
- Why is the research important?

Results:
- What does the experiment test?
- What method was used?
- What were the results?
- How do the data in the figure compare to what the authors write about the data in the text?

Discussion:
- What are the major conclusions of the research?
- Are the conclusions justified by the data presented?
- What would be the next logical step for the research project?

Sample Paper Study Guide

Paper: Spanogiannopoulos, P., Waglechner, N., Koteva, K., and Wright, G.D. (2014) A rifamycin inactivating phosphotransferase family shared by environmental and pathogenic bacteria. *Proc Natl Acad Sci* 11: 7102-7107

Introduction:
- How do rifamycin antibiotics work?
- What types of resistance mechanisms to rifamycin antibiotics are known and how do they prevent the antibiotic from working?
- What was known about genes encoding the enzyme that phosphorylates rifampin (RIF) before the work in this paper was done?

Results:
<u>For each part of each figure in the paper, diagram what experiment was done (if applicable). Explain what data are shown in the figure, and what controls were used. Use the questions below to guide your thinking about the results.</u>

Fig. 2A
- What is the RIF-associated element (RAE)?
- Why did the authors focus on the intergenic regions upstream of genes encoding known RIF-inactivating enzymes?
- What do the authors hypothesize about the role of the RAE?

Fig. 2B
- After identifying the RAE, what did the authors do next and why?
- What is the significance of the sequence LOGO in fig. 2B?

Fig. 2C
- Why did the authors compile ORFs that flank the RAE?
- What types of genes are associated with the RAE?

Fig. 3 (see also Fig. S1, S2 and Table S1- but we will focus on Fig. 3 in class)
- Why did the authors choose to focus on two strains *Streptomyces viridochromogenes* WAC4747 and *Streptomyces sviceus*?
- How and why did they choose the ORFs that they investigated?
- Why did the authors disrupt ORF1 and ORF2 from WAC4747?
- What data led the authors to believe that ORF1 but not ORF2 encodes the RIF phosphorylase enzyme, *rph*?

Table S2
- Why might the *rph* mutant strains be more sensitive to RIF than the wild-type strains?
- What possible explanation do the authors give to explain the MIC data?

Table 1 (top 4 datasets - pET22b, pET22b-*rph4747*, pET28b, pET28B-*rph-Ss*)
- What experiment was performed to give the data in Table 1?
- Do the results in Table 1 suggest that other genes from WAC4747 are needed in addition to *rpH* to provide RIF resistance? Explain your answer.

Fig. 4 and Fig. S3A,B
- How could the RAE DNA motif be involved in gene expression in response to RIF?

- What does it mean that transcript levels of *rph474* increase after growth in the presence of RIF? Does it mean that the RAE is responsible for the increase?
- What data suggest that the RAE is likely to be responsible for the increase in expression of *rph4747* in response to RIF?
    - Why was the RAE cloned upstream of the promoterless kan resistance gene?
    - Why is growth only seen around the RIF and RIF-SV discs?

Fig. S4 and Fig. 5
- Why did the authors compare the RPH4747 protein sequence to the sequence of PEP synthase?
- RPH4747 is annotated as a PEP synthase based on sequence alone, why would it not be a PEP synthase?
- How were proteins belonging to the RPH family identified?
- Because a protein is identified as being a member of the RPH family with this method, does it mean that the protein is definitely a RPH enzyme? Why or why not?
- What bacteria have genes that are members of the RPH family?
- Are all of the *rph* genes preceded by a RAE sequence? What is the significance of this?

Table 1 (bottom 4 datasets - pET19Tb, pET19TB-*rph-Bc*, pET19TB-*rph-Lm*)
- *B. cereus* and *L. monocytogenes* are susceptible to RIF. Do their rph genes confer RIF resistance to *E. coli*?
- What does this result mean? Does it make sense?

Fig. S6, S7, and Table S3
- Why biochemically characterize RPH activity given the other results in the paper (such as Fig. 3 and Table 1)?
- Name at least one piece of biochemical evidence showing that the purified RPH protein has the proposed activity - can phosphorylate RIF.
- What is the significance of the T-X-X-G-G-X-X-X-H motif? Why would mutating the H (histidine) to A (alanine) no longer make *E. coli* resistant to rif when the mutated *rph* gene was expressed in *E. coli*?

Discussion:
- Can you make a model for how the RAE would make expression of the *rph* gene responsive to RIF?
- What is a silent resistance gene and why do the authors put non-actinomycetes *rph* genes in this category?
- What experiment would you do next and why?

Glossary:

phylogeny- evolutionary tree

phylogenetics- study of evolutionary relatedness using sequencing data

heterologous protein expression- expressing a gene from one organism in a different organism

in vitro enzyme assays- biochemical experiments to test enzyme activity done outside of an organism

sequence logo- graphical representation of nucleotide or amino acid conservation, size of nucleotide indicates amount of conservation (bigger -> more conserved)

ORF- open reading frame, predicted protein coding sequence

orthologous genes or proteins- genes/proteins with homologous (similar) sequences that are related by speciation (similar sequences found in different species), generally assumed to have equivalent functions

BLAST- computer program for finding similar protein or nucleotide sequences from a database of nucleotide or protein sequences

contig- DNA segment generated as part of putting together genome sequences

upstream intergenic region- intergenic refers to regions in the DNA in between genes, upstream refers to those sequences 5' to the start of the gene, sequences that regulate gene expression are usually found in upstream intergenic regions near the promoter

HPLC chromatograms- high performance liquid chromatography is a method of column chromatography used to separate molecules based on a chemical characteristic, the chromatogram is the profile showing when molecules exit the column

quantitative RT-PCR- method to determine the amount of a specific RNA

conserved domain- functional or structural unit of a protein that is found in many different proteins, the domains are predicted to have a similar function

Conserved Domain Architecture Retrieval Tool- computer program that searches sequence databases to find proteins that have a similar set of domains in a particular order

cladogram- diagram showing evolutionary relatedness

clade- ancestor and all descendants of that ancestor

Advice On Giving A Research Talk

Structure of a Talk

Introduction:

- Start with the big picture- What is your area of research and why is it significant?
- Get more specific- present the system that you are investigating and how it relates to the big picture.
- Give any background that your audience needs to understand your research.
- Present the specific research question(s) that you are addressing.

Body of the talk:

- In this part you present your data - the results and conclusions of the experiments that you did to address the research question.
- If you are using a new method or a method that is not familiar to your audience, you can make a slide or part of a slide describing it. If you are using a common technique, you don't have to explain the method.

Conclusions:

- Go over the overall conclusion(s) from your experiments. Summarize what you have learned. Relate your conclusions back to the big picture.
- Optional - future directions

This outline is for a full research talk. You can modify this plan for your research talks to the class.

- If you don't have data, you can describe your overall research plan.
- If you have some data but no conclusions yet, you can present your data and describe what you plan to do next.
- If you have spent time trying to get something to work, you can explain what you did and how your tried to optimize the experiment.

Tips on making slides:

- Use 18 pt. font or larger.
- Keep the slide style consistent.
- Use colors that are easy to see. It is really hard to see yellow, for example.
- Don't make your presentation too busy with things like fancy slide transitions.
- Data slides (and most introductory slides) should have a heading that makes a statement about the subject of the slide, when possible. For ex. instead of "Role of MecA in Antibiotic Resistance" use "MecA Increases Resistance to Antibiotics".
- Use pictures, diagrams, and cartoons to describe things instead of words when possible.
- Use short bullet points and minimize the amount of text. You can explain the ideas more fully in your talk. Don't read your slides to the audience.
- Make one or at most 2 points per slide.

For more tips on giving a presentation check out:

From Uri Alon:

http://www.cell.com/molecular-cell/fulltext/S1097-2765(09)00742-4

From Susan McConnell at Stanford:

http://www.cellbio.duke.edu/faculty/research/Hogan.html (scroll down to the How to section on the lower right)

Oral Presentation Rubric

	poor	developing	acceptable	excellent
Title	no title	Title is wordy and unclear.<br>Title is not related to your work or is too broad.	Title is wordy but clear and related to your work.	Title is concise, clear, and describes your work well.
Introduction	absent	Background information lacks appropriate content for the listener to understand the scientific basis for the hypothesis or research question	Relevant background information is presented but not in a well organized manner. Hypothesis or research question does not logically follow from the background information.	Relevant background information is presented and organized such that the hypothesis or research question flow logically from it.
Hypothesis or Research Question	absent	A statement is made but is not a research question or a hypothesis.	Hypothesis or research question is stated but doesn't relate well to background information or was not clear and concise.	A clear and concise hypothesis or research question is presented that follows from the background information
Results	absent	Results do not relate to research question or hypothesis. Figures are confusing and methods are not explained.	Results are presented but figures are confusing. Methods are not explained well.	Results are presented in clear figures. Methods are explained. Experiments are related to the hypothesis and research question.
Conclusions	absent	Obvious conclusions are not identified.	Conclusions are unclear or not directly related to the results.	Clear and relevant conclusions are presented that are based on the results and directly connect to the hypothesis or research question.
References and Acknowledge-ments	absent	References are incomplete.<br>Not all contributors are acknowledged.	References are not all relevant or not all contributors are acknowledged.	References are cited when mentioned and people who contributed to the project are acknowledged
Presentation	No focus. No fore-thought given to talk.	Presentation is unclear and time is exceeded.	Presentation is not always clear.	Presentation is clear, well thought-out, and stays within the allotted time.

Adapted from Branchaw, J., Pfund, C., Rediske, R. Entering Research: A Facilitator's Manual 2010 W.H. Freeman and Company, New York, NY.

Presenter:______________________________

	poor	developing	acceptable	excellent	comments
Title					
Introduction					
Hypothesis/ Question					
Results					
Conclusions					
Ref./ Acknow-ledgements					
Presentation					
Additional comments					

Presenter:______________________________

	poor	developing	acceptable	excellent	comments
Title					
Introduction					
Hypothesis/ Question					
Results					
Conclusions					
Ref./Acknow-ledgements					
Presentation					
Additional comments					

POSTER TIPS

PARTS OF A POSTER

Title
- The title of your poster should be across the top of the poster in BIG font ≥54 pt.
- List the names and institutional affiliation of people who contributed to the work under the title.

Abstract
- Use the abstract that you submitted, unless the content of your poster has changed substantially.

Introduction
- Include a few panels (1-3 are best) that address the following questions. The goal of this part is to give enough general background about the system and the importance of your research question so that someone looking at the poster will know what you are doing and why.
  - What is the system?
  - Who are the key players?
  - What are you doing- what is the hypothesis or big question that you are addressing ?
  - Why is your work important?

Methods
- If your work involves a single method or if your research focuses on method development. Include this as a separate section. Otherwise you can integrate descriptions of the methods with the data panels.
- Use pictures when possible to illustrate a method.

Results
- Include one experiment per panel.
- Each result panel should have a title that summarizes the result in that panel.
- If you don't have a separate method panel include a legend that briefly describes the method.
- Make sure to label all axes and data series.
- Use graphs when possible to show data -choose appropriate graph type and axis type.
- Use text bullet points to describe key conclusions.

Conclusions
- Use bullet pointed text and/or a model figure (a picture/cartoon) describing what you have learned.

Future Directions
- Use bullet pointed text to describe what you will do next and why.

Acknowledgements
- Include all funding sources and help from non-authors.

VISUAL PRESENTATION

Font
- main text ≥24 pt.
- panel titles ≥ 36 pt.

POSTER TIPS

- poster title ≥ 54 pt. font
- Use an easy to read font like arial or helvetica

Colors
- Use a consistent color scheme throughout the poster.
- If you use the same strains, mutants, etc. in multiple experiments, always use the same color and symbols for each strain in all your result panels and model figures. For ex. wild type is always green squares.
- Make sure the colors are easily visible - don’t use yellow on a white background, for example.

Lines
- The axes and lines on your graphs must be thick enough to be seen well on the poster. They should be at least 1 pt. The Excel default is usually set to 0.75 pt.
- Borders and lines in figures and cartoons can be 2 pt or greater.

Pictures vs. Text
- When possible use a cartoon or other picture instead of text.
- When using text, use short bullet points, not long sentences and paragraphs (with the exception of the abstract).
- When importing a picture, make sure that the resolution is high enough so that the image is sharp. If the resolution is too low, the picture will be fuzzy.

Organization
- Make one major point per panel.
- Put panels into columns - each column should be viewed from top to bottom and columns viewed from left to right.

ORAL PRESENTATION

- Don’t read from your poster! Develop a short presentation that covers the main points of each panel, paraphrasing as needed. Focus on the main ideas, not the details. You can go over the details if someone is interested or asks. For the PSU undergraduate exhibition, you have 5 min. to present your poster to the judges.
- It is good to have two levels of presentation, one for an intelligent person who is not an expert in your general area of research and one for a life scientist who knows the basic of the science behind your research. Think of the first type of presentation as the NPR version or an explanation for a friend who is not majoring in life sciences. The second expert-level presentation can be more detailed.
- When presenting to non-scientists, think of metaphors or analogies that will help someone who doesn’t know what translation or genome sequencing is, for example, understand your work.
- Avoid using jargon that people outside of your lab will not understand.
- When you are talking about figures or graphs on your poster, point to the relevant part on the poster so people know what to look at.
- Start by introducing yourself. Say your name, the name of your research advisor, and the title of the poster.
- When you are presenting your poster, look at the people listening. If they look confused, ask if they have any questions.

POSTER TIPS

- Don’t forget to talk about why the research that you are doing is important!
- Practice!!! Practice!!! Practice!!!
- Dress well. You don’t have to wear a suit, but don’t wear a grubby t-shirt and jeans.
- When you are finished, ask if anyone has any questions.
- Thank the people listening for coming to your poster. You can either do this at the beginning before you start or at the end when you are finished.
